# Supplementary material for: Pathogenic neurofibromatosis type 1 (NF1) RNA splicing resolved by targeted RNAseq
Source: NPJ Genom Med. 2021 Nov 15;6:95. doi: 10.1038/s41525-021-00258-w (PMC8593033; doi:10.1038/s41525-021-00258-w)
Supplement: Supplementary file 1 — Supplementary Information [file 41525_2021_258_MOESM1_ESM.pdf]

Supplementary information for

# Pathogenic Neurofibromatosis type 1 (NF1) RNA splicing resolved by targeted RNAseq

R. Koster[1], R.D. Brandão[1,2], D. Tserpelis[1], C.E.P. van Roozendaal[1], C.N. van Oosterhoud[1], K.B.M. Claes[4], A.D.C. Paulussen[1,2], M. Sinnema[1], M. Vreeburg[1], V. van der Schoot[1], C.T.R.M. Stumpel[1], M.P.G. Broen[5], L. Spruijt[6], M.C.J. Jongmans[8,9], S.A.J. Lesnik Oberstein[10], A.S. Plomp[11], M. Misra-Isrie[11], F.A. Duijkers[12], M.J. Louwers[13], R. Szklarczyk[1], K.W.J. Derks[1], H.G. Brunner[1,2,3,6,7], A. van den Wijngaard[1], M. van Geel[1,2], M.J. Blok[1,2#]

#Corresponding Author:
M.J. Blok, Ph.D.
rien.blok@mumc.nl

Supplementary information includes:

**Supplementary Figure 1.** Targeted RNAseq-based analysis to detect normal and pathogenic *NF1* RNA-splicing

**Supplementary Figure 2**. Top QURNAS events for validation sample 4 and 5 (both WT-NF1) are artefacts.

**Supplementary Figure 3**. *In silico* predictions for effect c.586+5G>A (left) and observed changes in splicing for validation sample 6.

**Supplementary Figure 4.** *In silico* predictions for effect c.1466A>G (p.Tyr489Cys) and observed changes in splicing for validation sample 7.

**Supplementary Figure 5.** ERS values reference exon–exon splice junctions for validation of SureSelect capture.

**Supplementary Figure 6.** Enrichment score of normal *SPRED1* exon-exon splice junctions of the undetermined cohort.

**Supplementary figure 7.** Undiagnosed sample 1.

**Supplementary Figure 8.** Undiagnosed sample 2.

**Supplementary Figure 9.** Undiagnosed sample 5.

**Supplementary Figure 10.** Undiagnosed sample 6.

**Supplementary Figure 11.** Undiagnosed sample 8.

**Supplementary Data** files:

- **Supplementary Data 1.** Raw QURNAs output of validation samples
- **Supplementary Data 2.** Detected splicing events validation, replication and molecular undiagnosed cases
- **Supplementary Data 3.** Published normal splicing events
- **Supplementary Data 4.** Raw QURNAs output of replication samples
- **Supplementary Data 5.** Raw QURNAs output of molecular undiagnosed cases

**Supplementary Figure 1.** Targeted RNAseq-based analysis to detect normal and pathogenic *NF1* RNA-splicing

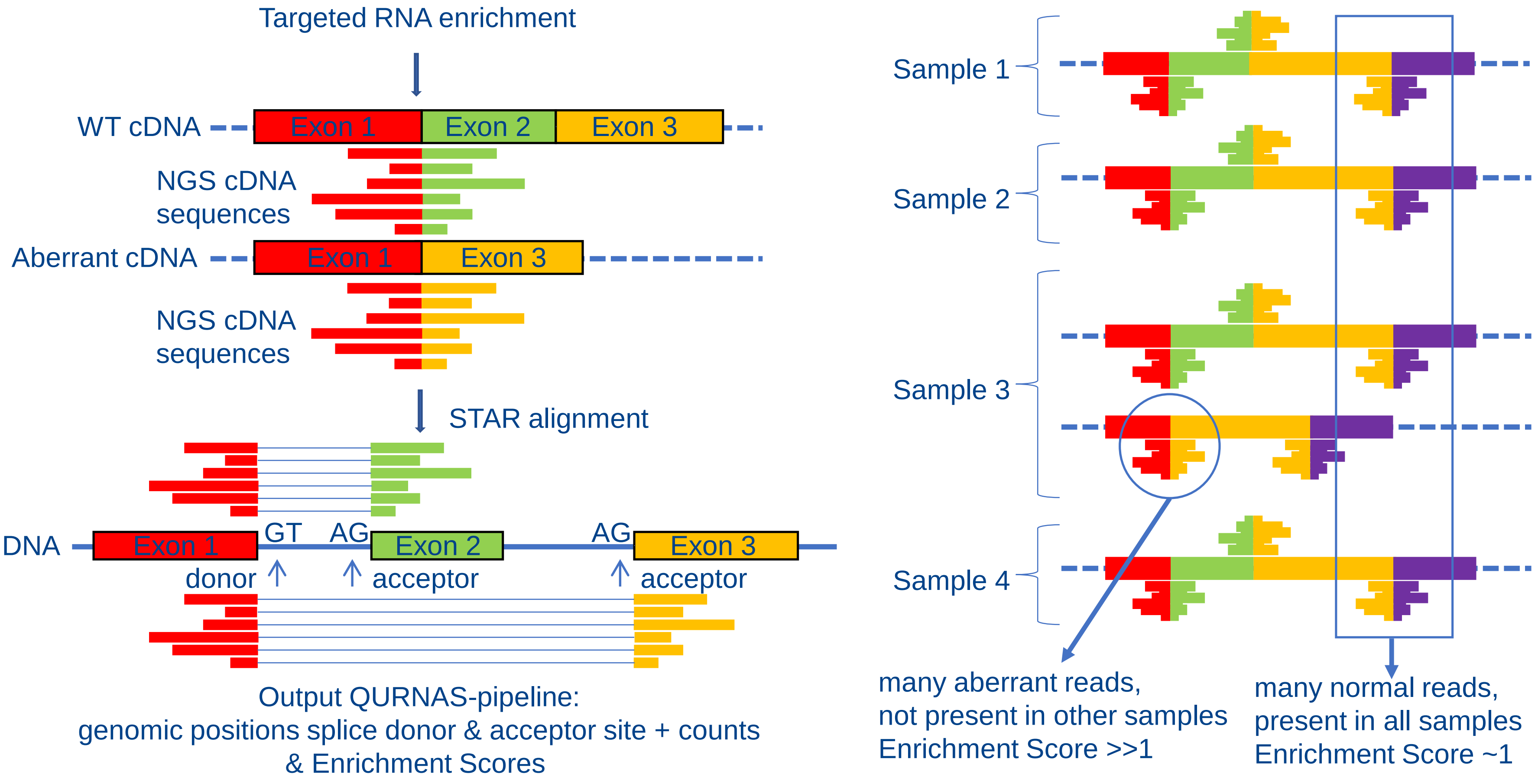


| Genomic position | | Enrichment Score (ERS) | | | | |
|---|---|---|---|---|---|---|
| Donor site | Acceptor site | Sample 1 | Sample 2 | Sample 3 | Sample 4 | |
| 29527612 | 29528054 | 1.0 | 1.2 | **0.6** | 0.9 | Exon 1-2 junction |
| 29527612 | 29528428 | 0 | 0 | **18** | 0 | Exon 2 skip |
| 29528176 | 29528428 | 0.9 | 1.1 | **0.7** | 1.0 | Exon 2-3 junction |
| 29528502 | 29533257 | 1.1 | 1.0 | 1.0 | 0.9 | Exon 3-4 junction |

# **Supplementary Figure 2.** Top QURNAS events for validation sample 4 and 5 (both WT-NF1) are artefacts.

**Validation sample 4** (WT)

Highest event: deletion (Δ) 5'exon 37 - 5’exon 41, c. 4986_6506del; ERS = 5; 150 reads

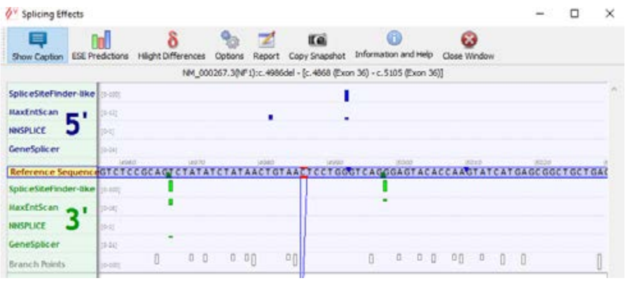


Exon 37, supposed splicing start, no *in silico* predictions for a splice donor site at this position

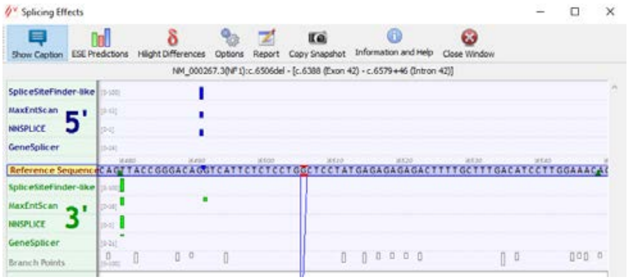


Exon 41, supposed splicing stop , no *in silico* predictions for a splice acceptor site at this position

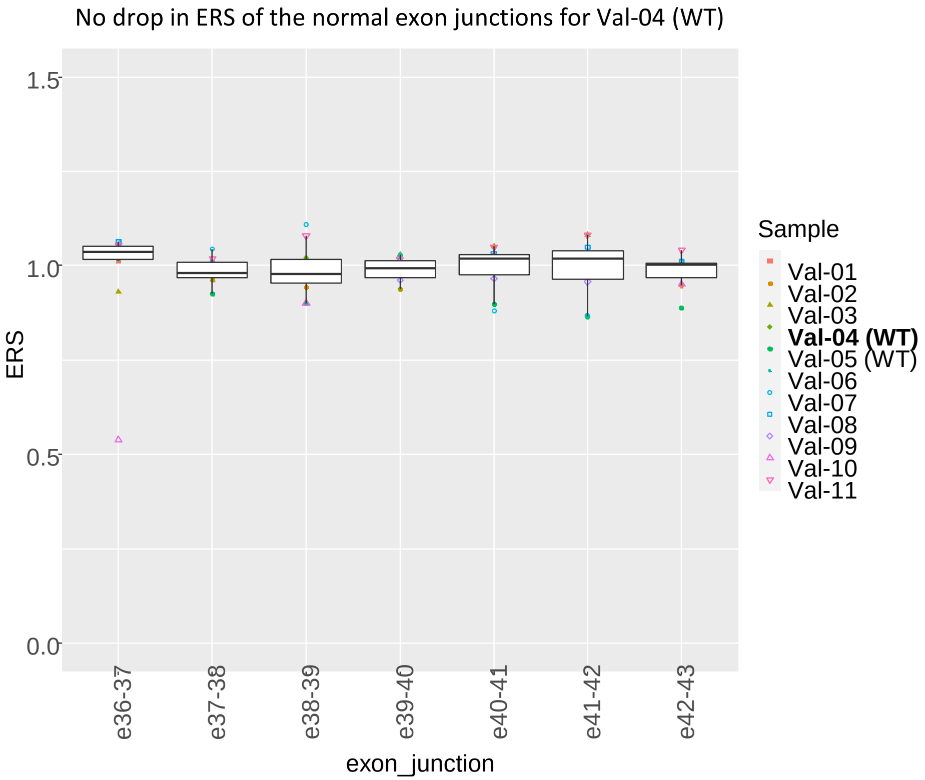


**Validation sample 5** (WT)

Δ 3’UTR c.-85_-68del; ERS 6.7, 90 reads

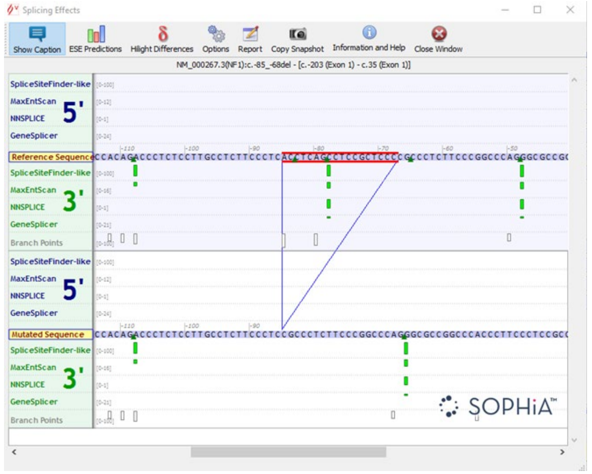


3’UTR, supposed splicing start/stop , no *in silico* predictions for a splice donor/acceptor at these positions => small deletion/artefact

**Supplementary Figure 3.** *In silico* predictions for effect c.586+5G>A (left) and observed changes in splicing for validation sample 6, versus median values across all samples in the run (right).

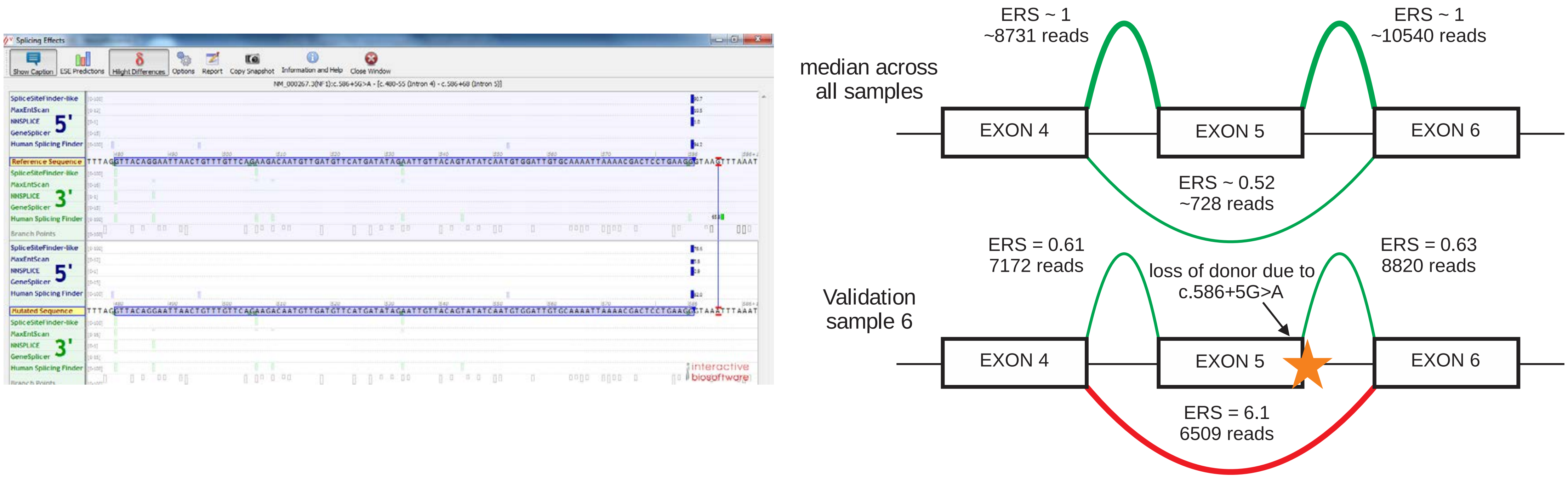

**Supplementary Figure 4.** *In silico* predictions for effect c.1466A>G (p.Tyr489Cys) and observed changes in splicing for validation sample 7 versus median values across all samples in the run (left). The QURNAS top event, Δ45nt exon 18 (IF) is an artefact (right).

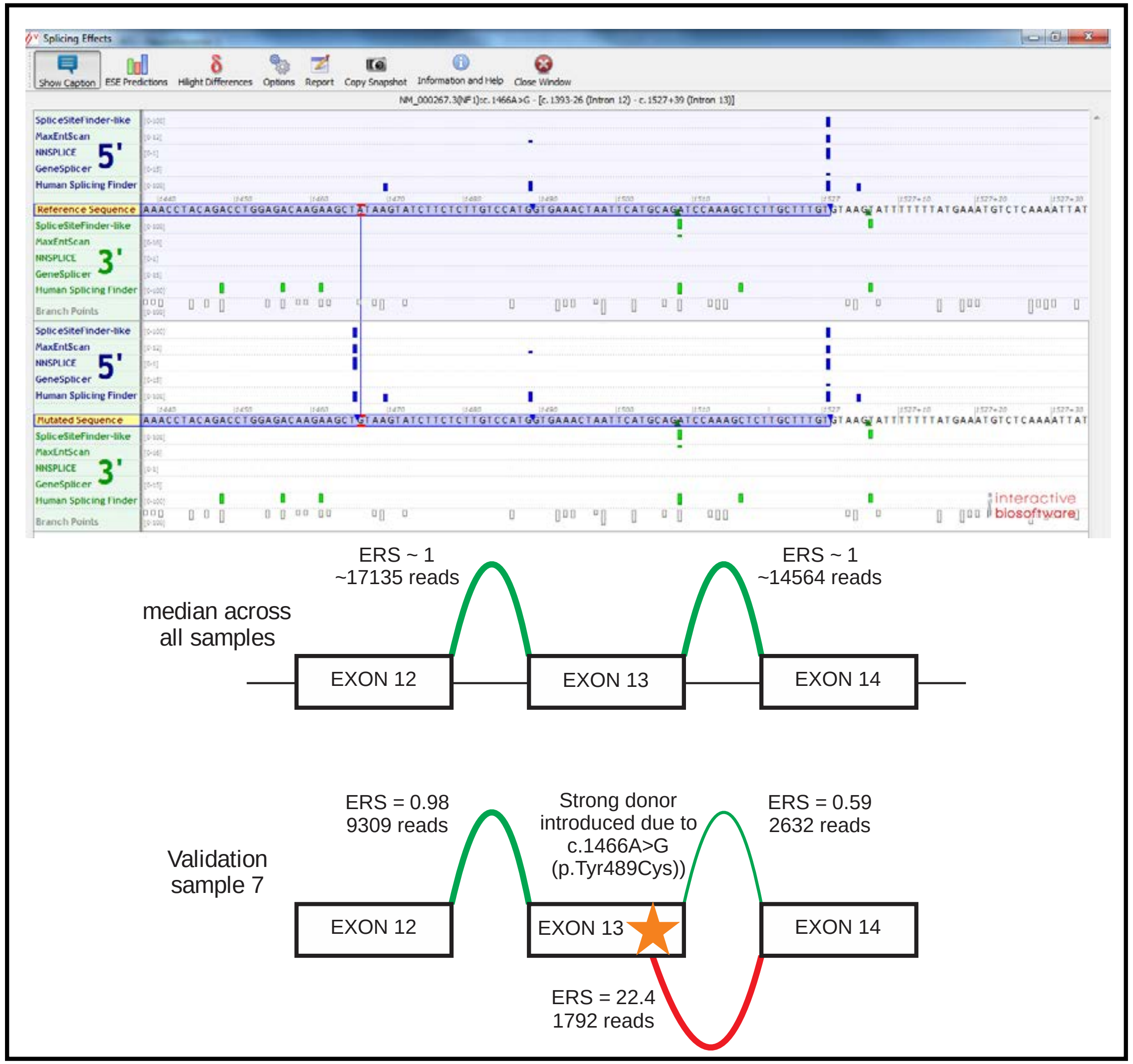


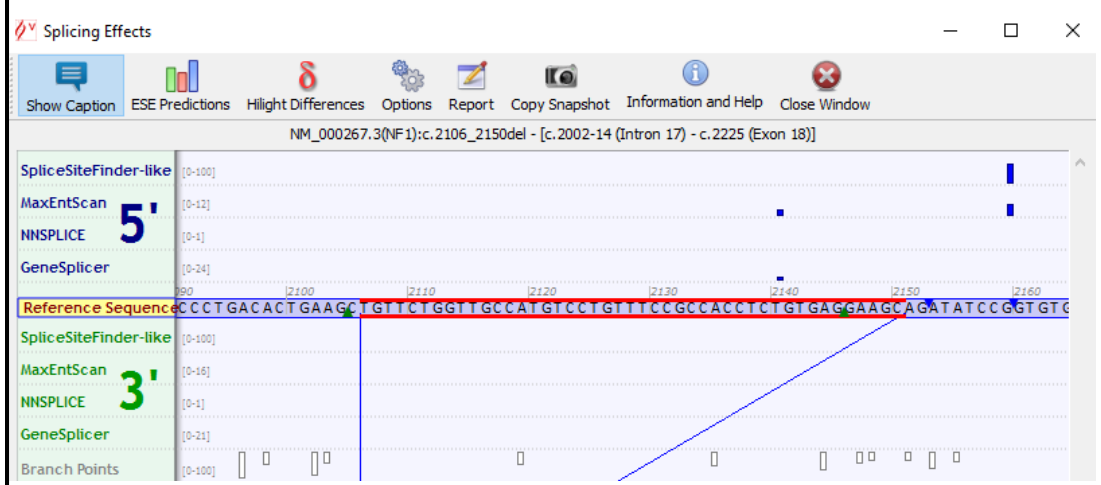


The Δ45nt are depicted in red. Note that no acceptor nor donor site are predicted that could explain the splicing of these 45nt.

**Supplementary Figure 5.** ERS values reference exon–exon splice junctions. Validation SureSelect capture using part of the replication samples.

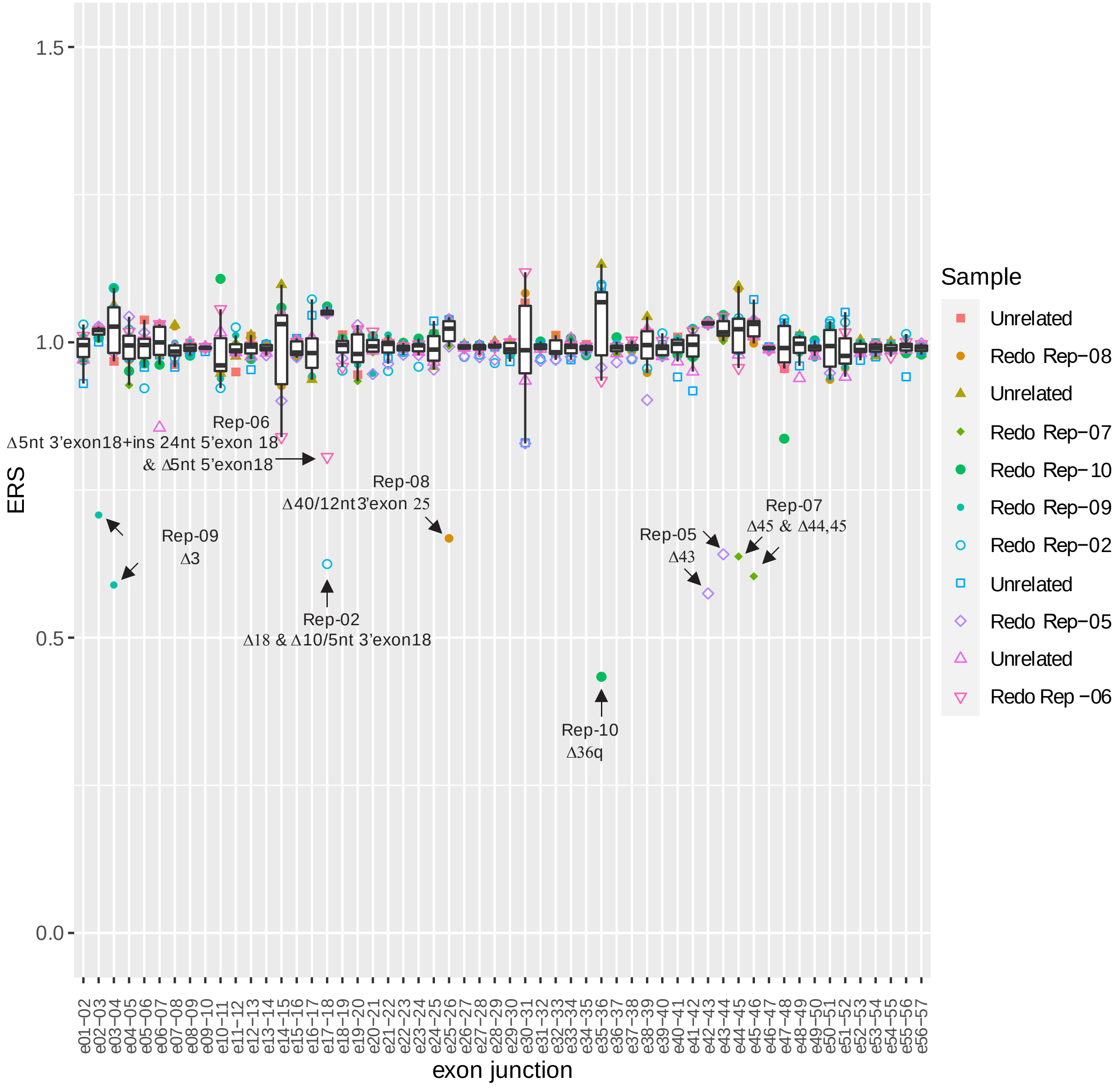

**Supplementary Figure 6.** Enrichment score of normal *SPRED1* exon-exon splice junctions of the undetermined cohort .

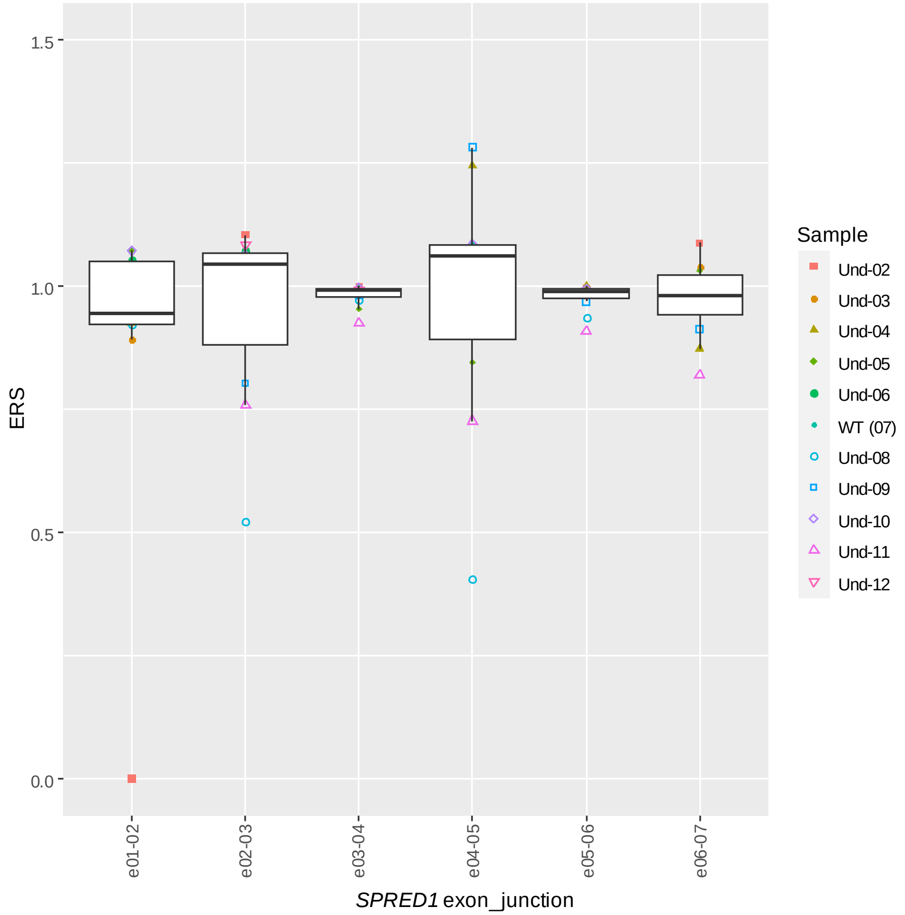


Und-02 shows unexplained bi-allelic loss of the WT exon 1-2 junction. This samples has a pathogenic *NF1* variant c.6580-2A>G. A bi-allelic *SPRED1* splicing variant is therefore not likely. Und-08 shows outliers because of low sample quality.

**Supplementary figure 7.** Undiagnosed sample 1 - c.5749+332A>G. This variant can be observed in the cDNA reads in a genome browser (IGV). Modest allelic imbalance allele RNA versus DNA sequence reads (left). *In silico* predictions splice effect for the c.5749+332A>G variant (right).

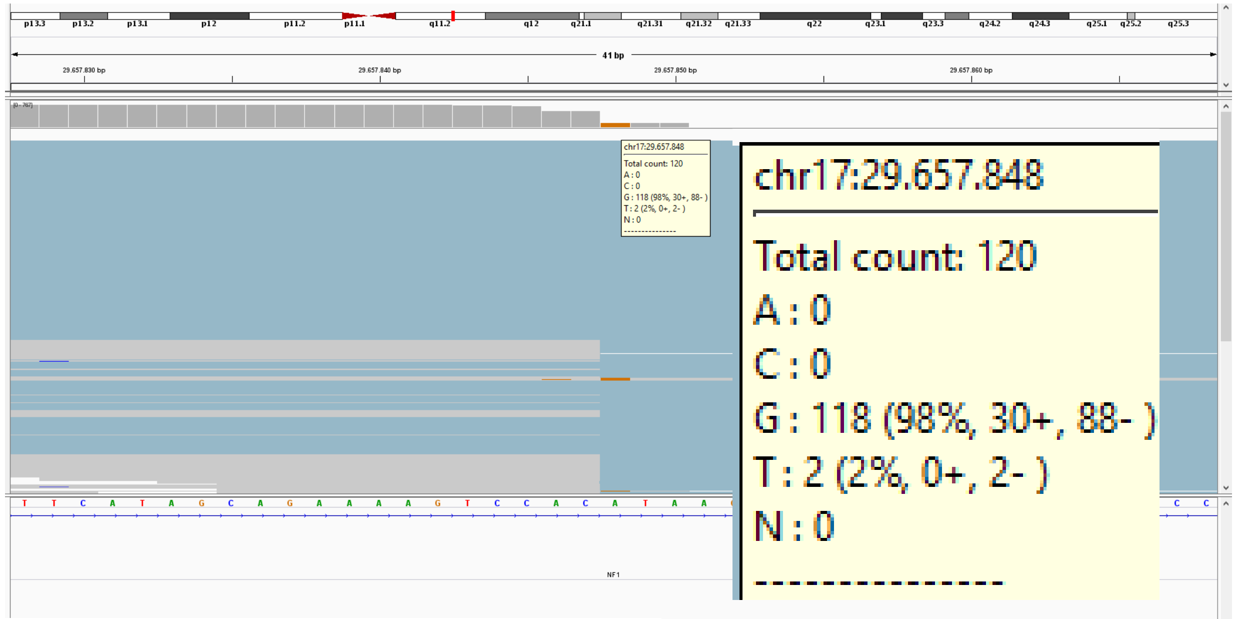


| Variant | DNA | RNA |
|---|---|---|
| c.5749+332A>G | N/A | G 98% |
| c.702G>A | A 49% | A 41% |
| c.2034G>A | A 52% | A 30% |
| c.*2265C>G | G 49% | G 31% |
| c.*2829T>C | C 51% | C 29% |

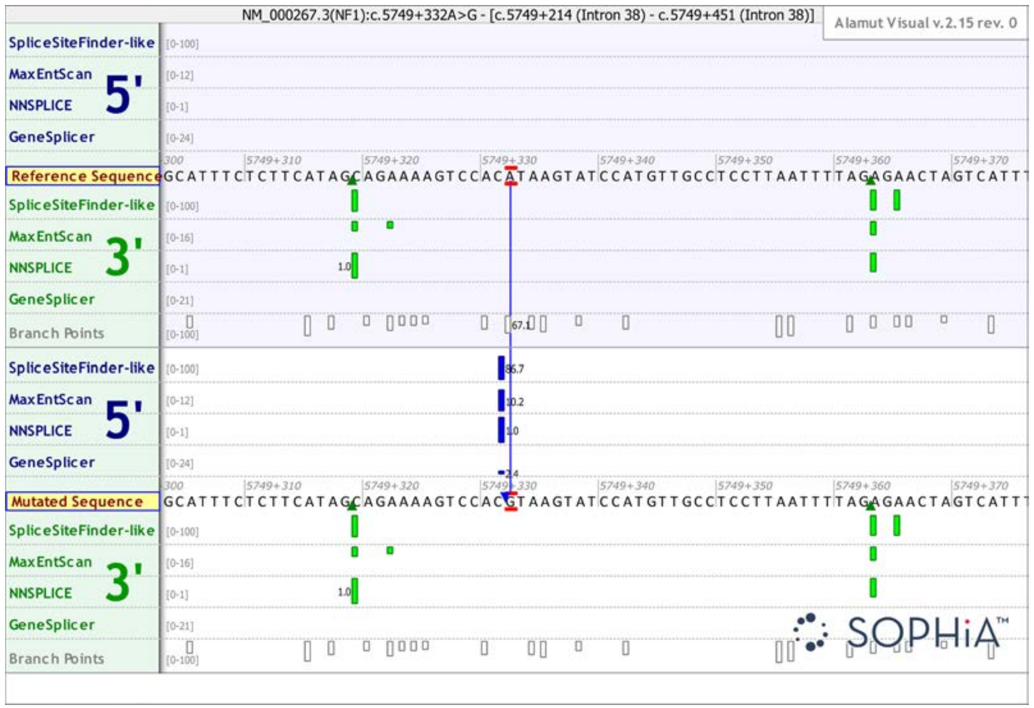

**Supplementary Figure 8.** Undiagnosed sample 2; *In silico* predictions for c.6580-2A>G (left) and observed changes in splicing for sample 2 versus median values across all samples in the run (right).

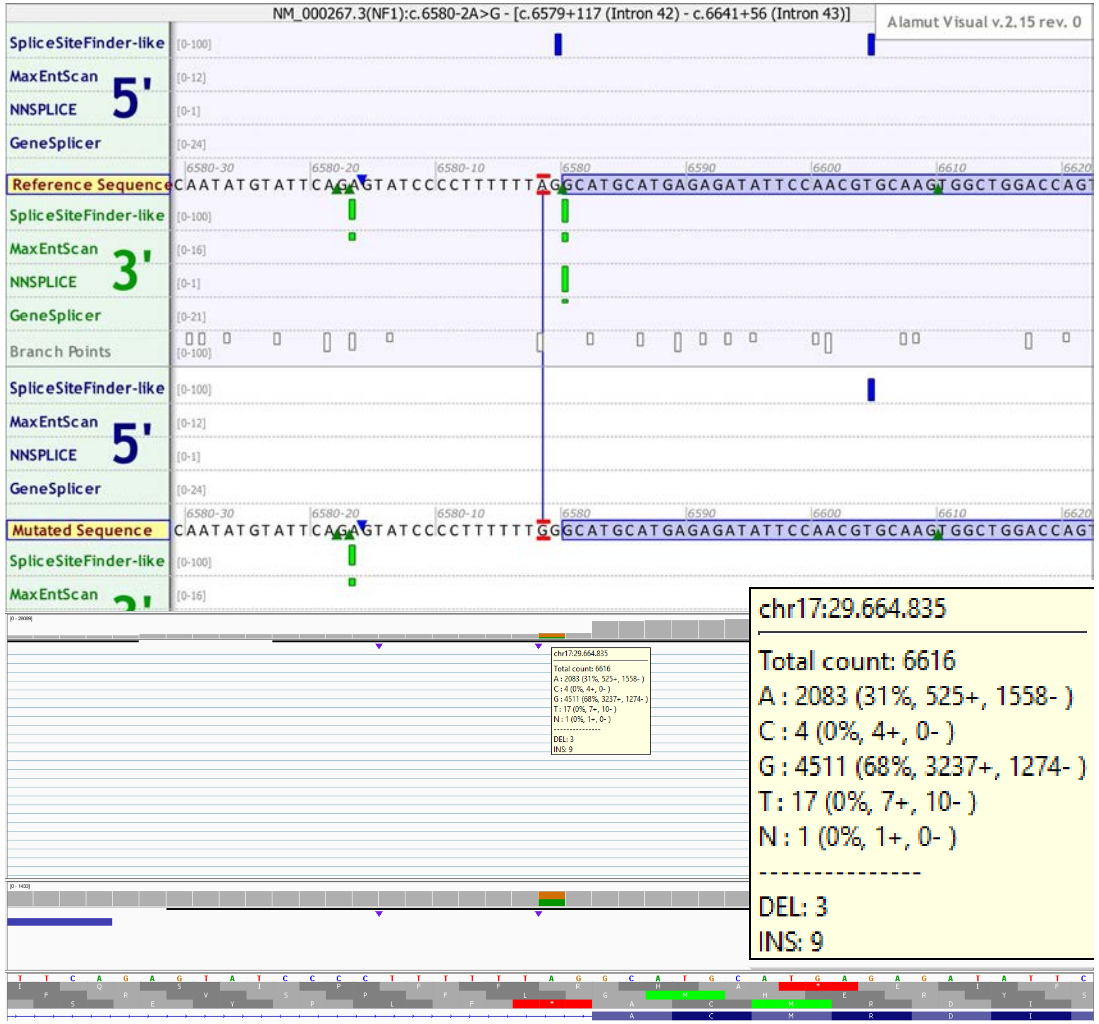


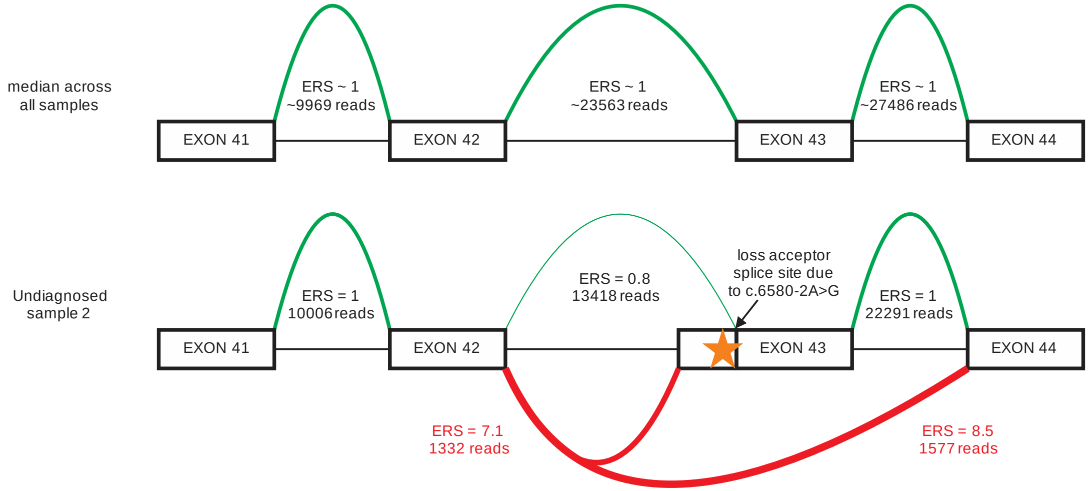

**Supplementary Figure 9.** Undiagnosed sample 5; *In silico* predictions for c.1260+1604A>G and observed changes in splicing versus median values across all samples in the run (left). Modest allelic imbalance allele RNA versus DNA sequence reads (right).

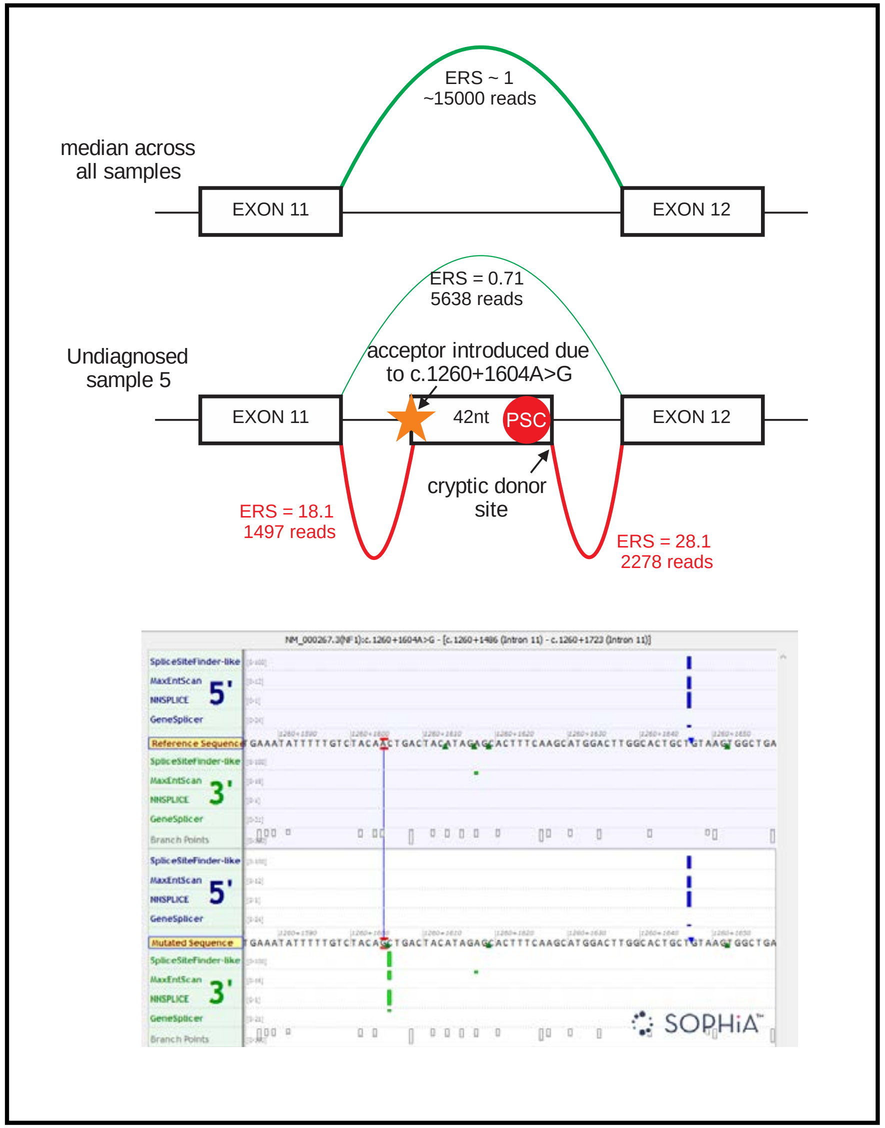


NM_000267.3(NF1):c.1260+1604A>G

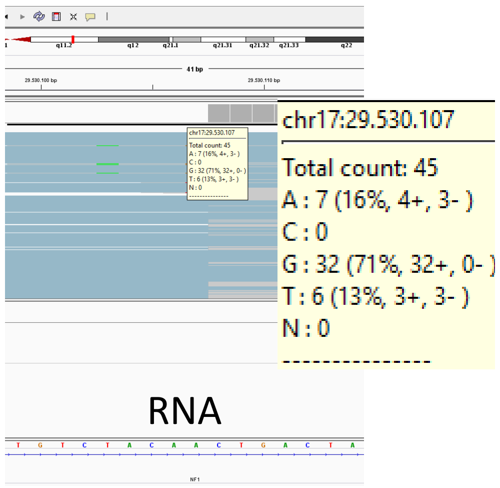


| Variant | DNA | RNA |
|---|---|---|
| c.1260+1604A>G | N/A | G 71% |
| c.168C>T | T 54% | T 35% |
| c.702G>A | A 49% | A 34% |
| c.2034G>A | A 49% | A 64% |
| c.*2829T>C | C 54% | C 36% |

NM_000267.3(NF1):c.*2829T>C

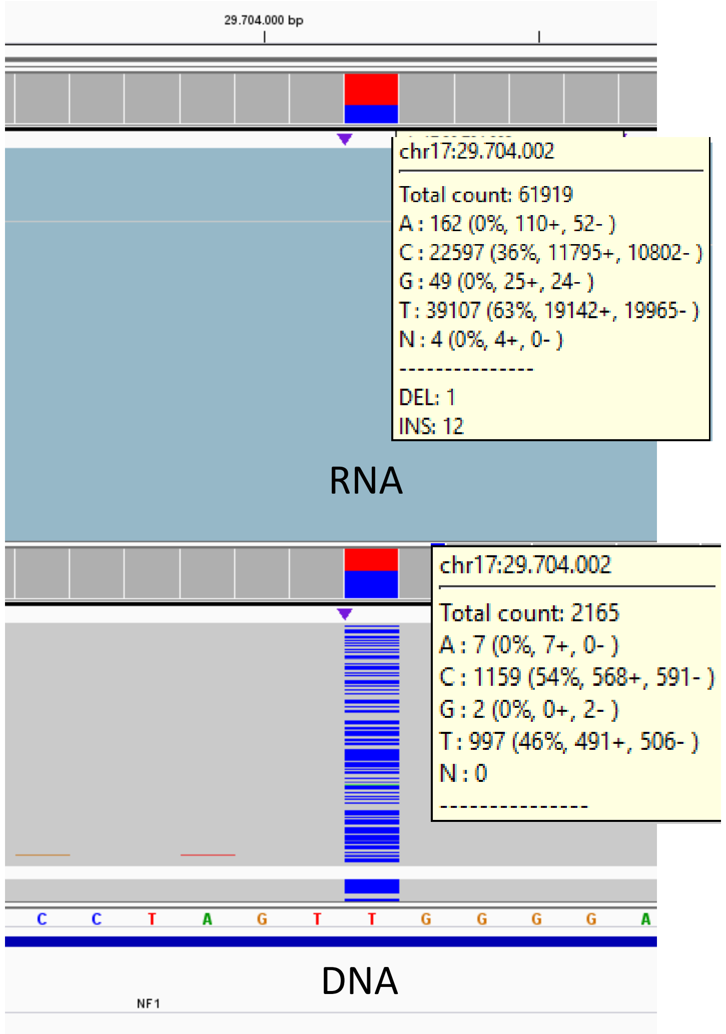

**Supplementary Figure 10.** Undiagnosed sample 6; *In silico* predictions for c.2252G>T (p.(Gly751Val)) and observed changes in splicing versus median values across all samples in the run (left). Strong allelic imbalance in RNA versus DNA sequence reads (right).

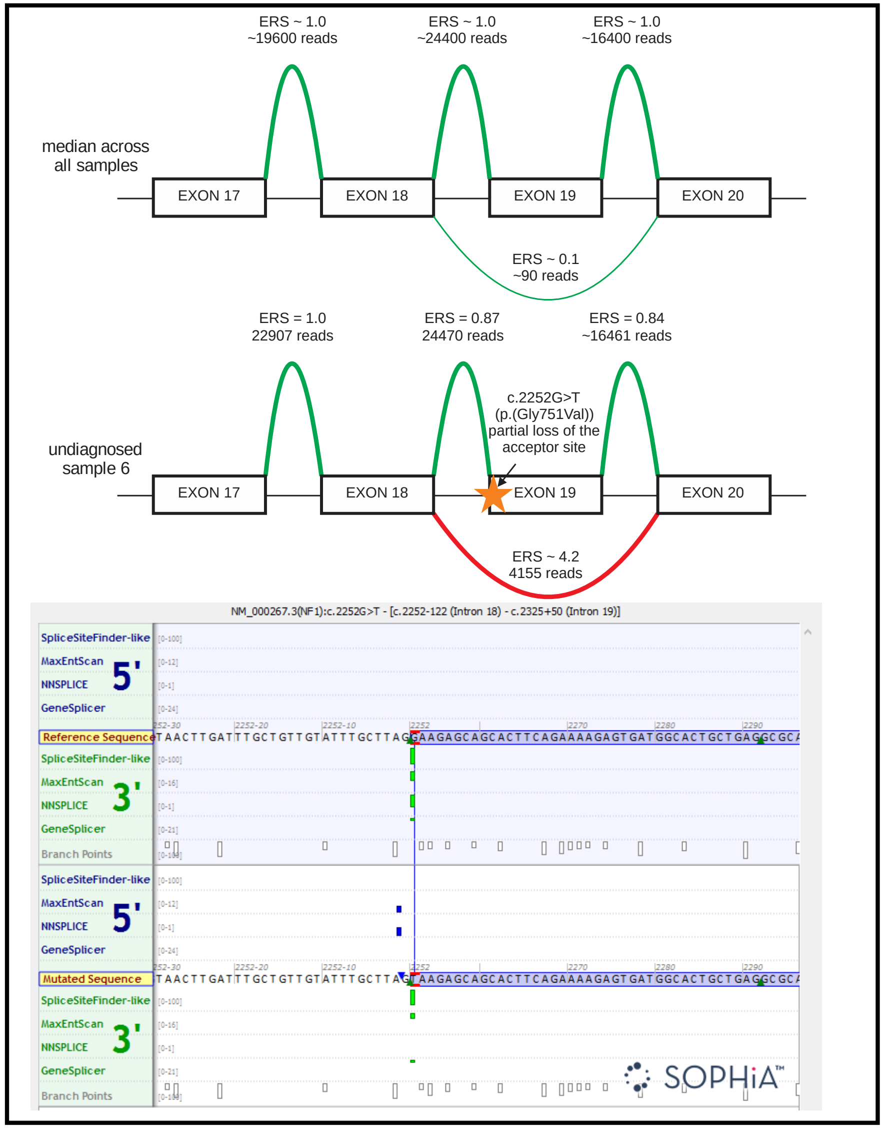


NM_000267.3(NF1):c.2252G>T

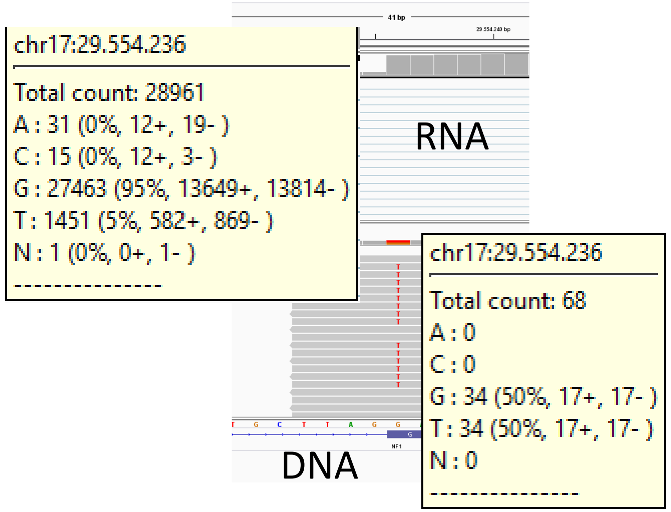


NM_000267.3(NF1):c.*2829T>C

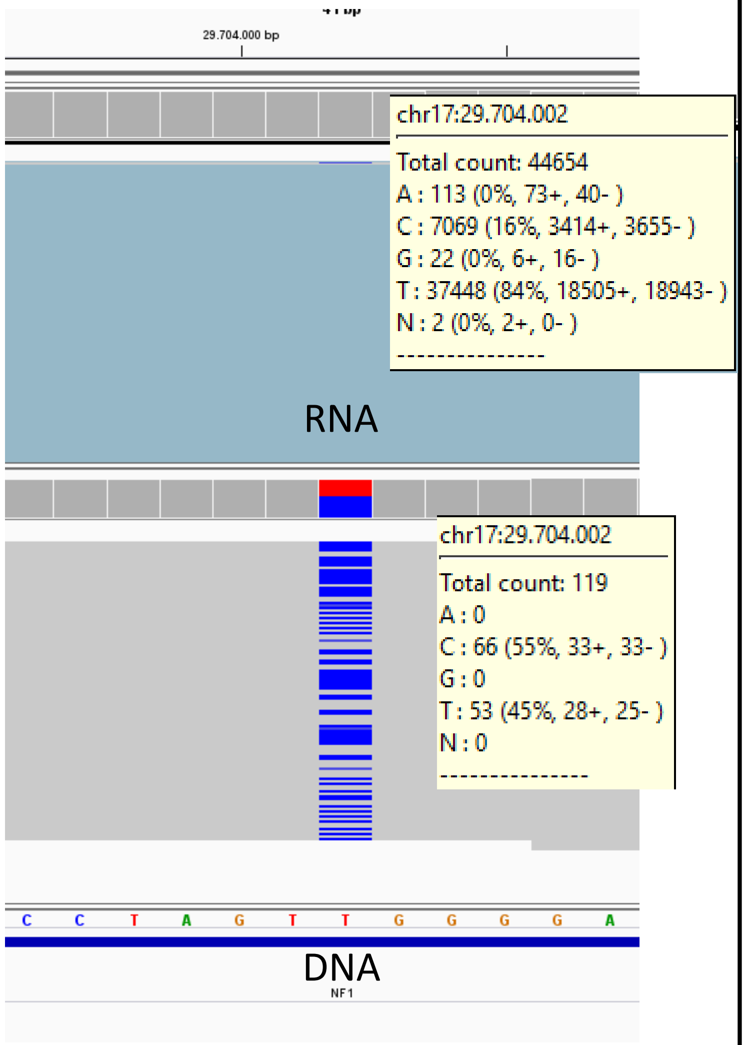


| Variant | DNA | RNA |
|---|---|---|
| c.2252G>T | T 50% | T 5% |
| c.702G>A | A 61% | A 23% |
| c.2034G>A | A 44% | A 79% |
| c.*2829T>C | C 55% | C 16% |

**Supplementary Figure 11.** Undiagnosed sample 8; *In silico* predictions for c.556G>T (p.(Asp186Tyr)) and observed changes in splicing versus median values across all samples in the run (left). Weak allelic imbalance in RNA versus DNA sequence reads (right).

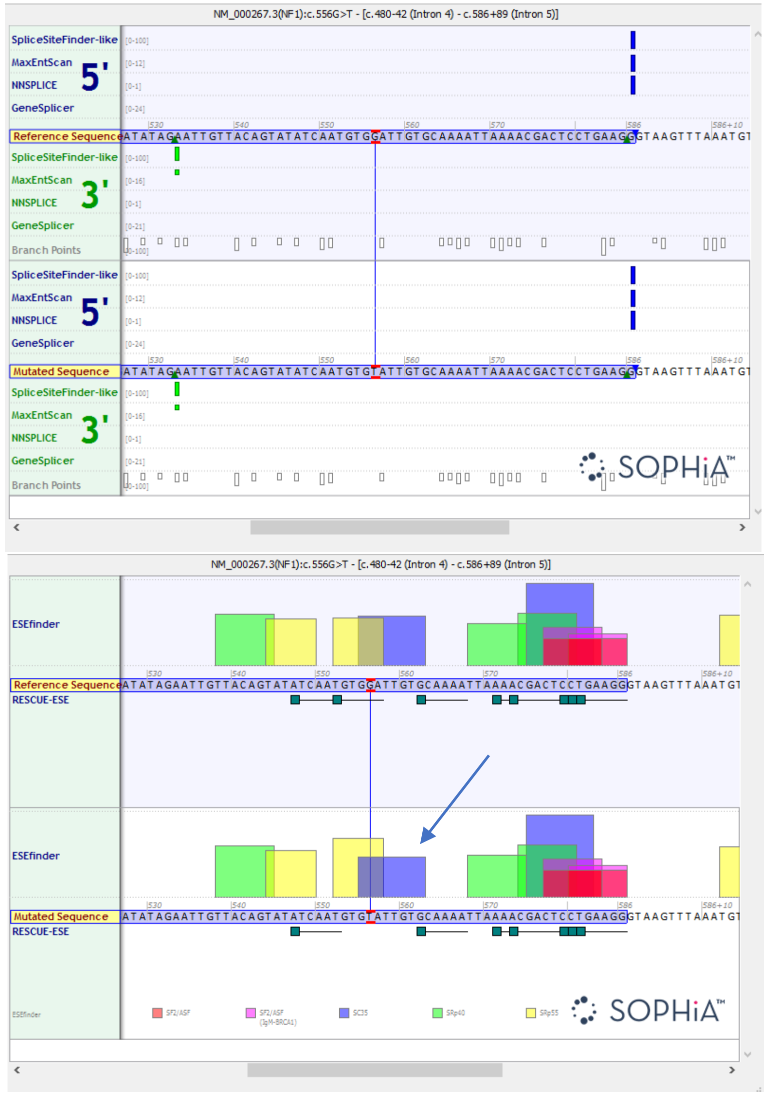


NM_000267.3(NF1):c.556G>T

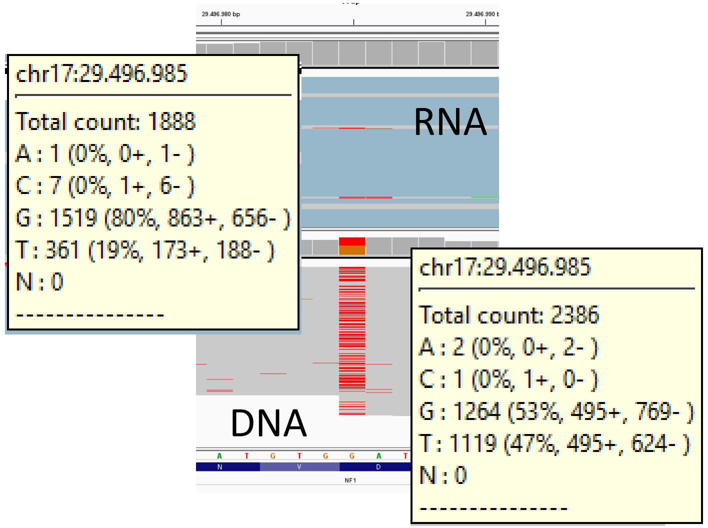


NM_000267.3(NF1):c.*2201G>A

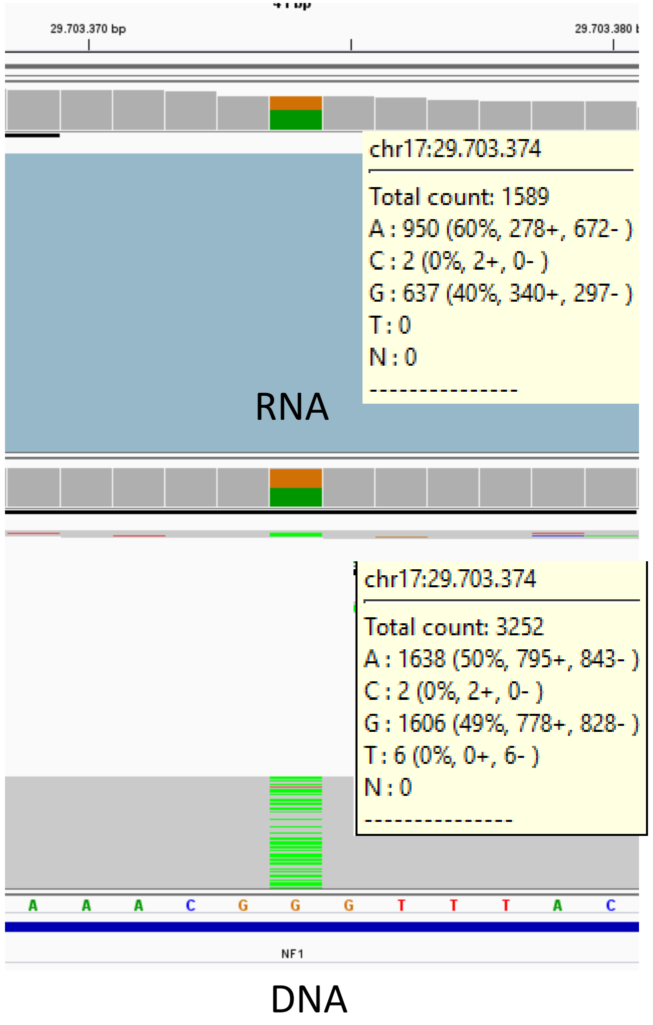


| Variant | DNA | RNA |
|---|---|---|
| c.556G>T | T 47% | T 19% |
| c.702G>A | A 48% | A 35% |
| c.2034G>A | A 50% | A 50% |
| c.*2201G>A | A 50% | A 60% |
