# Supplementary material for: Pathogenic neurofibromatosis type 1 (NF1) RNA splicing resolved by targeted RNAseq
Source: NPJ Genom Med. 2021 Nov 15;6:95. doi: 10.1038/s41525-021-00258-w (PMC8593033; doi:10.1038/s41525-021-00258-w)
Supplement: Supplementary file 7 — Reporting Summary [file 41525_2021_258_MOESM7_ESM.pdf]

nature portfolio

Corresponding author(s): Rien Blok, PhD

Last updated by author(s): Aug 9, 2021

Reporting Summary

Nature Portfolio wishes to improve the reproducibility of the work that we publish. This form provides structure for consistency and transparency in reporting. For further information on Nature Portfolio policies, see our Editorial Policies and the Editorial Policy Checklist.

Statistics

For all statistical analyses, confirm that the following items are present in the figure legend, table legend, main text, or Methods section.

n/a	Confirmed	
☒	☐	The exact sample size (n) for each experimental group/condition, given as a discrete number and unit of measurement
☒	☐	A statement on whether measurements were taken from distinct samples or whether the same sample was measured repeatedly
☒	☐	The statistical test(s) used AND whether they are one- or two-sided<br>*Only common tests should be described solely by name; describe more complex techniques in the Methods section.*
☒	☐	A description of all covariates tested
☒	☐	A description of any assumptions or corrections, such as tests of normality and adjustment for multiple comparisons
☒	☐	A full description of the statistical parameters including central tendency (e.g. means) or other basic estimates (e.g. regression coefficient) AND variation (e.g. standard deviation) or associated estimates of uncertainty (e.g. confidence intervals)
☒	☐	For null hypothesis testing, the test statistic (e.g. F, t, r) with confidence intervals, effect sizes, degrees of freedom and P value noted<br>*Give P values as exact values whenever suitable.*
☒	☐	For Bayesian analysis, information on the choice of priors and Markov chain Monte Carlo settings
☒	☐	For hierarchical and complex designs, identification of the appropriate level for tests and full reporting of outcomes
☒	☐	Estimates of effect sizes (e.g. Cohen's d, Pearson's r), indicating how they were calculated

Our web collection on statistics for biologists contains articles on many of the points above.

Software and code

Policy information about availability of computer code

Data collection	*Provide a description of all commercial, open source and custom code used to collect the data in this study, specifying the version used OR state that no software was used.*
Data analysis	STAR v2.4.1d for sequence alignment to the reference genome, QURNAs in-house made tool for splice site detection (available at https://dataverse.nl/dataset.xhtml?persistentId=hdl:10411/LY8ZQ4) , Alamutv2.15 for variant assesment, IGV v2.4.18 for bam visualization

For manuscripts utilizing custom algorithms or software that are central to the research but not yet described in published literature, software must be made available to editors and reviewers. We strongly encourage code deposition in a community repository (e.g. GitHub). See the Nature Portfolio guidelines for submitting code & software for further information.

Data

Policy information about availability of data

All manuscripts must include a data availability statement. This statement should provide the following information, where applicable:

- Accession codes, unique identifiers, or web links for publicly available datasets
- A description of any restrictions on data availability
- For clinical datasets or third party data, please ensure that the statement adheres to our policy

The authors confirm that all data, except aligned sequence reads, supporting the findings of this study are available within the article and/or its supplementary materials. Aligned sequences reads (BAM files) are deposited at the European Variant Archive under accession code EGAD00001007978.

Field-specific reporting

Please select the one below that is the best fit for your research. If you are not sure, read the appropriate sections before making your selection.

☒ Life sciences ☐ Behavioural & social sciences ☐ Ecological, evolutionary & environmental sciences

For a reference copy of the document with all sections, see nature.com/documents/nr-reporting-summary-flat.pdf

Life sciences study design

All studies must disclose on these points even when the disclosure is negative.

Sample size	proof of principle study; using 21 NF1 patients with known NF1 splicing variants to show that the technique works and 11 previously undiagnosed patients NF1 with potential splicing variants
Data exclusions	proof of principle study, data analysis does not rely on using statisics
Replication	proof of principle study, data analysis does not rely on using statisics
Randomization	proof of principle study, data analysis does not rely on using statisics, furtermore not an intervention study therefore randomization is not applicable
Blinding	proof of principle study, data analysis does not rely on using statisics, furtermore not an intervention study therefore randomization/blinding is not

Reporting for specific materials, systems and methods

We require information from authors about some types of materials, experimental systems and methods used in many studies. Here, indicate whether each material, system or method listed is relevant to your study. If you are not sure if a list item applies to your research, read the appropriate section before selecting a response.

Materials & experimental systems

n/a	Involved in the study	
☒	☐	Antibodies
☒	☐	Eukaryotic cell lines
☒	☐	Palaeontology and archaeology
☒	☐	Animals and other organisms
☐	☒	Human research participants
☒	☐	Clinical data
☒	☐	Dual use research of concern

Methods

n/a	Involved in the study	
☒	☐	ChIP-seq
☒	☐	Flow cytometry
☒	☐	MRI-based neuroimaging

Human research participants

Policy information about studies involving human research participants

Population characteristics	NF1 disease with proven or suspected splicing variants.
Recruitment	The samples used in this study were from patients undergoing diagnostic genetic testing.
Ethics oversight	The institutional Medical Ethics Committee (medisch-ethische toetsingscommissie or METC) of the Maastricht University Medical Center confirmed that the Medical Research Involving Human Subjects Act (WMO) does not apply to our study (METC 2021-2666) and that an official approval of this study by the committee is not required.

Note that full information on the approval of the study protocol must also be provided in the manuscript.
